# Determination of Binding Sites on Trastuzumab and Pertuzumab to Selective Affimers Using Hydrogen–Deuterium Exchange Mass Spectrometry

**DOI:** 10.1021/jasms.3c00069

**Published:** 2023-03-24

**Authors:** Oladapo Olaleye, Christian Graf, Baubek Spanov, Natalia Govorukhina, Matthew R. Groves, Nico C. van de Merbel, Rainer Bischoff

**Affiliations:** † Analytical Biochemistry, Department of Pharmacy, University of Groningen, A. Deusinglaan 1, 9713 AV Groningen, The Netherlands; ‡ Novartis Technical Research & Development Biologics, Hexal AG, Keltenring 1 + 3, 82041 Oberhaching, Germany; § Drug Design, Department of Pharmacy, University of Groningen, A. Deusinglaan 1, 9713 AV Groningen, The Netherlands; ∥ ICON Bioanalytical Laboratories, Amerikaweg 18, 9407 TK Assen, The Netherlands

## Abstract

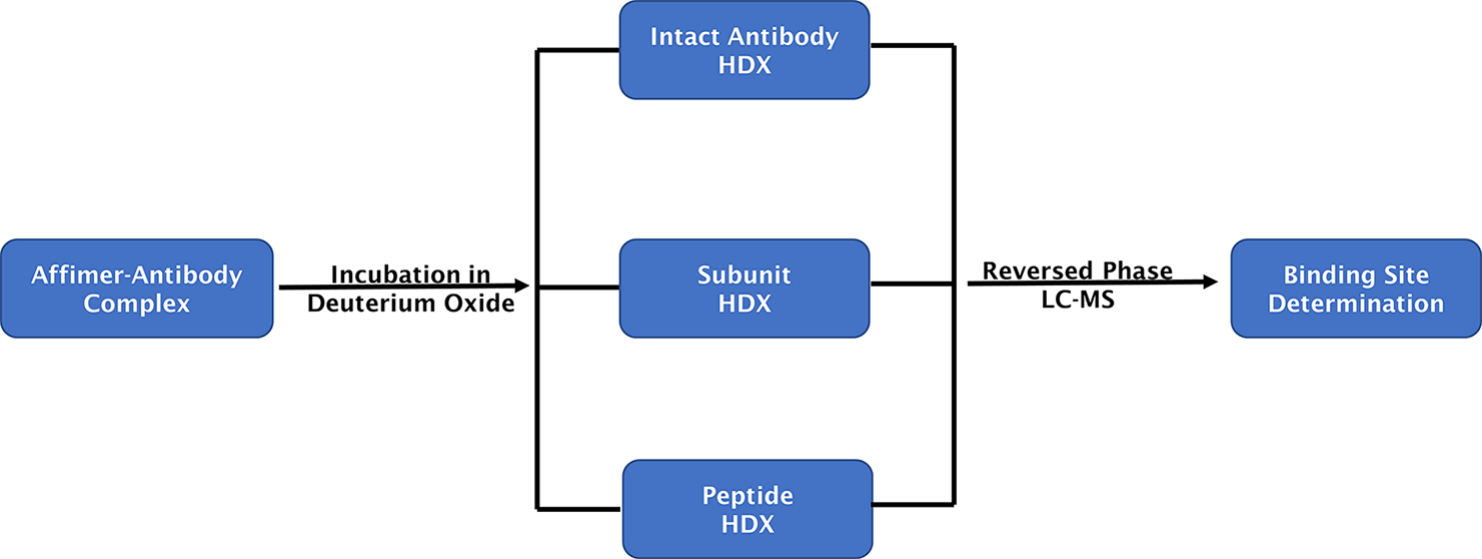

Hydrogen–deuterium exchange mass spectrometry (HDX-MS) is a method to probe the solvent accessibility and conformational dynamics of a protein or a protein–ligand complex with respect to exchangeable amide hydrogens. Here, we present the application of HDX-MS to determine the binding sites of Affimer reagents to the monoclonal antibodies trastuzumab and pertuzumab, respectively. Intact and subunit level HDX-MS analysis of antibody-affimer complexes showed significant protection from HDX in the antibody Fab region upon affimer binding. Bottom-up HDX-MS experiments including online pepsin digestion revealed that the binding sites of the affimer reagents were mainly located in the complementarity-determining region (CDR) 2 of the heavy chain of the respective antibodies. Three-dimensional models of the binding interaction between the affimer reagents and the antibodies were built by homology modeling and molecular docking based on the HDX data.

## Introduction

Binding site mapping is an important step in the characterization of an affinity reagent that should bind a target protein selectively.^1^ A binding site, or an epitope in the case of antibodies, is an area on the target protein to which a binding partner or antibody binds,^2^ and the mapping process involves the determination of this area. Epitopes can be described as linear epitopes (continuous epitopes), which remain functional even after a protein has been denatured or digested into peptides, and nonlinear epitopes (also known as discontinuous or conformational epitopes) that are only functional in correctly folded proteins or large folded protein fragments.^3,4^ Binding site mapping is useful in improving the understanding of the immune response and autoimmunity, in obtaining appropriate antigens for vaccine production, in defining antibody specificity and mechanism of action,^5^ in assay development, and in understanding the fundamental aspects of protein–protein interactions.^1^

Different approaches have been used in binding site mapping. X-ray crystallography is referred to as the gold standard^4,6,7^ because it gives a detailed image of the interaction at atomic resolution. This approach requires that high-quality crystals of the complex are generated and then subjected to X-ray diffraction.^4^ The need for high-purity proteins and the challenge of finding suitable conditions to obtain high-quality crystals make X-ray crystallography labor-intensive and time-consuming in addition to requiring a high level of expertise.^5,8^ Site-directed mutagenesis is an approach to binding site mapping that involves the binding of an affinity ligand to mutated forms of a target protein.^5^ Large numbers of mutated forms can be quickly screened, and loss of binding indicates that a mutation is in the associated binding site.^4^ A disadvantage of this technique is that false positives can occur because mutations may result in changes in protein structure that affect the binding site indirectly.^1,9^ NMR is another binding site mapping technique that provides a dynamic image of the interaction between the binding partner and target protein in solution.^4^ NMR requires stable-isotope labeling (e.g., ^15^N or ^13^C) and prior determination of the target protein structure.^5^ The NMR spectrum of the free target protein is then compared to the spectrum of the target protein when in a complex with the unlabeled affinity binder. Changes in the NMR spectrum indicate the area on the target protein that was affected by binding.^10^ NMR is generally limited to proteins up to 35 kDa.^3,4,8^ Peptide-based approaches for binding site mapping involve the immobilization of overlapping peptides covering the entire target protein sequence on solid surfaces. An enzyme-linked immunosorbent assay (ELISA) is used to determine the binding site after exposure to the binding partner.^5^ Exposure of the peptides to the binding partner can be performed using peptide arrays,^11^ phage display libraries,^12^ or synthetic peptide libraries.^13^ Peptide-based approaches provide a rapid way of screening large numbers of possible binding sites,^5^ but their application is limited to linear epitopes, since nonlinear, conformational epitopes cannot be mimicked by linear peptides.^4,5,10^ There is also a chance of false positives, because of highly hydrophobic peptides, which may bind nonspecifically to the binding partner,^5^ but the use of appropriate negative controls helps to avoid this caveat.^14^ Limited proteolysis coupled to mass spectrometry (MS) is another approach for binding site mapping. In this method, a specific protease that cleaves the target protein at the epitope and nonrelated parts is employed.^1^ A comparison of the free target protein to the complex after cleavage with the protease is used to determine the binding site.^15^ A disadvantage of limited proteolysis is that it requires the presence of protease-specific cleavage sites at appropriate locations in the target protein sequence^1^ and that the complex must remain intact during proteolysis.

Hydrogen–deuterium exchange mass spectrometry (HDX-MS) is based on the exchange of hydrogen for deuterium atoms in amide hydrogens of the polypeptide backbone leading to an increase in mass. Measurement of mass increments or shifts introduced by deuterium exchange is then followed by MS, usually in high-resolution mode.^16−20^ A main challenge of HDX-MS is that deuterium atoms will rapidly back-exchange when brought into contact with water, for example during chromatographic peptide separation.^21,22^ That is why it is preferred to use fully automated systems that work under highly reproducible conditions.^23,24^ In addition to binding site mapping, HDX-MS can also be used for studying protein aggregation,^25^ in the characterization of biopharmaceuticals in terms of structural stability,^26^ protein structure–function analysis,^27^ and protein–protein complex analysis.^28^ However, HDX-MS is unable to provide the atomic resolution or three-dimensional structure information confered by X-ray crystallography or NMR^7,8^ but can provide useful information for molecular modeling and simulations.^29^

Trastuzumab and pertuzumab are monoclonal antibodies for the treatment of patients with human epidermal growth factor receptor-2 (HER2) positive breast cancer with an increase in overall survival upon treatment.^30−32^ Mass spectrometry is a key method for the structural characterization of monoclonal antibodies to analyze structural integrity and post-translational modifications. Several analytical strategies are now routinely applied to assess the antibody primary structure with increased resolution, including top-down or intact, middle-up, and bottom-up approaches^33^ using specific enzymatic digestion.

Affimer reagents are a group of binding proteins based on the human protease inhibitor stefin A or phytocystatin protein scaffolds. They can be selected to specifically bind different targets using phage display technology.^34^ Affimer reagents have been used for the specific enrichment of these therapeutic antibodies from blood plasma by Olaleye et al.^35^ It was thus of interest to elucidate the binding sites that allowed to achieve specificity.

Here, we describe an HDX-MS approach for mapping the binding sites on trastuzumab and pertuzumab to these Affimer reagents. HDX-MS analysis of preformed protein–protein complexes was performed at the intact antibody, subunit, and peptide level. The obtained results formed the basis for modeling the three-dimensional structure of the binding regions of both antibodies to the respective Affimer reagents. An important focus was to understand the susceptibility of the residues in the binding sites to undergo modifications. Modifications of residues in binding sites on a target have been reported to result in loss of recognition of a binder.^36^ Therefore, determining the binding sites could provide information on any possible modifications of the residues that may prevent the Affimer reagents from binding to the antibodies.

## Materials and Methods

Trastuzumab (Herceptin, Lot. no. N7185H03) and pertuzumab (Perjeta, Lot. no. H0319H03) were purchased from Roche (Almere, The Netherlands). Antitrastuzumab and antipertuzumab Affimer reagents (antitrastuzumab 386_737_A7 and antipertuzumab 00557_709097) were produced and supplied by Avacta Life Sciences (Wetherby, U.K.). BioWhittaker Dulbecco’s phosphate-buffered saline (DPBS; 1×; cat. no. 17-512F) was purchased from Lonza (Walkersville, MA, USA). Deuterium oxide (D_2_O; CAS. no. 7789-20-0) was purchased from Cambridge Isotope Laboratories (Andover, MA, USA). FabRICATOR enzyme (IdeS; cat. no. A0-FR1-096) was obtained from Genovis AB (Lund, Sweden). Formic acid (FA; cat. no. 94318-250mL) and trifluoroacetic acid (TFA; cat. no. T6508-100ML) were obtained from Honeywell (Offenbach am Main, Germany). Guanidinium hydrochloride (GdnCl; 8 M; cat. no. 50937-100mL), hydrochloric acid (HCl ≥ 37%; cat. no. 30721-1L), and Tris(2-carboxyethyl)phosphine hydrochloride (TCEP; cat. no. 75259-10G) were purchased from Sigma-Aldrich (Zwijndrecht, The Netherlands). Sodium hydroxide solution (1 M NaOH; Pr. no. 1.09137.1000), dipotassium hydrogen phosphate (K_2_HPO_4_; Pr. no. 1.05101.1000), potassium dihydrogen phosphate (KH_2_PO_4_; Pr. no. 1.04873.1000), acetonitrile (ACN; Pr. no. 1.00030.2500), and Amicon Ultra Centrifugal Filter Units (0.5 mL; Ultracel 30 kDa; cat. no. UFC503096) were obtained from Merck (Darmstadt, Germany).

### Preparation of Trastuzumab and Pertuzumab Fragment Antibody Binding [F(ab′)_2_] and Fragment Cystallizable (Fc/2) Subunits

3 mg/mL of intact antibodies in 100 μL of PBS was incubated with 300 units of lyophilized IdeS in a ratio of 1 μg of antibody to 1 unit of IdeS for 60 min at 37 °C.

### Preparation of Affimer Reagent–Antibody and −F(ab′)_2_ Complexes

3 mg/mL of intact antibodies and F(ab′)_2_ forms in 50 μL of PBS were incubated in 325 μL containing 2 mg/mL of Affimer reagents (in PBS) in the ratio of 1:5 (mass to mass) for 90 min at room temperature. To remove the excess unbound Affimer reagents, the resulting solution was transferred to a 30 kDa Amicon Ultra centrifugal filter unit. The volume in the unit was adjusted to 500 μL with PBS. The filter unit was centrifuged at 8385 rcf for 3 min, and the flow-through was discarded. The addition of PBS and subsequent centrifugation were repeated twice to remove unbound Affimer reagents.

### Hydrogen–Deuterium Exchange (HDX)

HDX was performed on a LEAP HDx-3 PAL platform robot operated with the Chronos software (Trajan Scientific and Medical, Milton Keynes, U.K.). For the intact and subunit level experiments, 3 μL of free intact antibodies and F(ab′)_2_ subunits as well as Affimer reagent–antibody and −F(ab′)_2_ complexes were incubated in 57 μL of labeling buffer (5 mM K_2_HPO_4_ and 5 mM KH_2_PO_4_ in D_2_O, pH7) for 60, 300, and 600 s (in triplicates). 50 μL of the deuterated samples was mixed with 50 μL of quench buffer (50 mM K_2_HPO_4_, 50 mM KH_2_PO_4_, in water, pH 2.3 adjusted with 1 M NaOH) at 0 °C to stop the HDX reaction. Control samples (both free and complexed forms) were incubated in equilibration buffer (5 mM K_2_HPO_4_ and 5 mM KH_2_PO_4_ in water, pH 7) before mixing with quench buffer. 50 μL of the sample-quench buffer mixture was then injected for LC-MS analysis (Graf et al., unpublished data).

For the peptide level experiments, 3 μL of free intact antibodies and F(ab′)_2_ subunits, as well as Affimer reagent–antibody and −F(ab′)_2_ complexes, were incubated in 57 μL of labeling buffer (5 mM K_2_HPO_4_ and 5 mM KH_2_PO_4_ in D_2_O, pH 7) for 300 and 600 s (five replicates). Next, 50 μL of the deuterated samples was mixed with 50 μL of quench buffer (50 mM K_2_HPO_4_, 50 mM KH_2_PO_4_, 4 M GdnCl, and 500 mM TCEP in water, pH 2.3 adjusted with 1 M NaOH) at 0 °C to stop HDX. Control samples (both free and complexed forms) were incubated in equilibration buffer (5 mM K_2_HPO_4_ and 5 mM KH_2_PO_4_ in water, pH 7 adjusted with 1 M HCl) before mixing with quench buffer (in triplicates). 50 μL of the sample-quench buffer mixture was then digested with an in-line pepsin column at 20 °C before LC-MS analysis (see the following paragraph for details).

### LC-MS Analysis

MS data were acquired using a system consisting of a Nano ACQUITY/M-Class UPLC (Binary and Auxiliary pumps), HDX Manager, and a SYNAPT G2-S quadrupole–ion mobility–time of flight mass spectrometer (Waters, Milford, MA, USA).

For the intact and subunit sample analysis, proteins are desalted for 2 min and chromatographically separated at 0 °C using a Bioresolve RP mAb Polyphenyl VanGuard Cartridge (2.7 μm, 450 Å pore size, 2.1 × 5 mm, part. no. 186008943, Waters, Milford, MA, USA). Mobile phase A was 10% ACN, 0.1% FA, and 0.02% TFA in water, and mobile phase B was 10% H_2_O, 0.1% FA, and 0.02% TFA in ACN. The binary pump ran at 70 μL/min with a 6 min linear gradient from 0 to 90% B, after which the column was cleaned (2 min at 90% B) and equilibrated (1.5 min at 0% B).

For peptide analysis, digestion was performed in line with the LC-MS using an Enzymate BEH pepsin column (2.1 × 30 mm, part no. 186007233, Waters, Milford, MA, USA) via the auxiliary pump at 70 μL/min of 100% A and at 20 °C. After trapping with an ACQUITY UPLC BEH C18 Vanguard Precolumn (1.7 μm, 130 Å pore size, 2.1 × 5 mm, part. no. 186003975, Waters, Milford, MA, USA) for 3 min, chromatographic separation was performed at 0 °C using an ACQUITY UPLC BEH C18 analytical column (1.7 μm, 130 Å pore size, 1.0 × 100 mm, part. no. 186002346, Waters, Milford, MA, USA) Mobile phase A was 0.2% FA in water, and mobile phase B was 0.2% FA in ACN. The binary pump ran at 40 μL/min with a 6 min linear gradient from 5 to 35% B and then 35–40% B for 1 min, after which the column was cleaned (2 min at 95% B) and equilibrated (2 min at 5% B).

MS analysis was performed using the following conditions: ESI positive, capillary voltage 3.0 kV, cone voltage (120 V for intact, 90 V for F(ab′)_2_, and 30 V for peptides), source offset 50 V, source temperature 90 °C, desolvation gas 800L/h, and nebulizer gas 6.0 bar. Calibration of the instrument was performed using sodium iodide for intact masses (mass range 400–4000 *m*/*z*) and sodium formate for peptide masses (mass range 260–2000 *m*/*z*). For peptide analysis, MS/MS settings had an *m*/*z* range of 50–2000 and scan time of 0.3 s to obtain both precursor and fragment ion data simultaneously by alternating the collision energy between low (4 eV) and high (ramp 18–40 eV) values. Leucine enkephalin solution (*m*/*z* 556.2771) at a concentration of 50 pg/μL (in 50:50 ACN/H_2_O, 0.1% formic acid) was infused at a flow rate of 20 μL/min via a lock spray interface to improve mass accuracy. The MS system was operated under the Waters MassLynx software suite (version 4.2).

### Data Analysis

Average masses of unlabeled or labeled intact F(ab′)_2_ and fragment crystallizable (Fc/2) subunits of trastuzumab and pertuzumab were processed after deconvolution of MS spectra generated in the intact and F(ab′)_2_ experiments. The deconvolution was performed using the IntactMass protein module of BYOS Desktop Software (Version 4.1, Protein Metrics, Cupertino, CA, USA) or using the MaxEnt1 algorithm from MassLynx 4.2 software. Deuterium incorporation into intact and subunit proteins was determined by calculating the difference between labeled and unlabeled proteins: Δ*D* (difference in deuterium incorporation) = average mass (*x* s HDX) – average mass (0 s HDX). The processed data were exported for visualization in GraphPad Prism 8 (GraphPad Software, Inc., San Diego, CA, USA).

MS data from the peptide experiments were processed using ProteinLynx Global Server (PLGS, Version 3.0.2, Waters, Milford, MA, USA), HDExaminer (Version 3.3, Sierra Analytics, Modesto, CA, USA), and DynamX HDX Data Analysis Software (Version 3.0, Waters, Milford, MA, USA).

### Modeling of Binding Regions

Affimer reagent structures were created using SWISS-MODEL^37^ after searching for an appropriate template using their amino acid sequences. The selected affimer template was from the Protein Data Bank (PDB, http://www.rcsb.org/; accession number 5ML9). Trastuzumab and pertuzumab F(ab′)_2_ structures were obtained from the Protein Data Bank (PDB, http://www.rcsb.org/ accession number; 6OGE). Binding regions were modeled using HADDOCK 2.4^38,39^ after defining the residues on both antibodies likely involved in the binding process from the HDX data. The resulting models were then exported into the PyMOL Molecular Graphics System (Version 2.5.2, Schrödinger, New York, USA) for visualization.

## Results and Discussion

**Figure 1:**
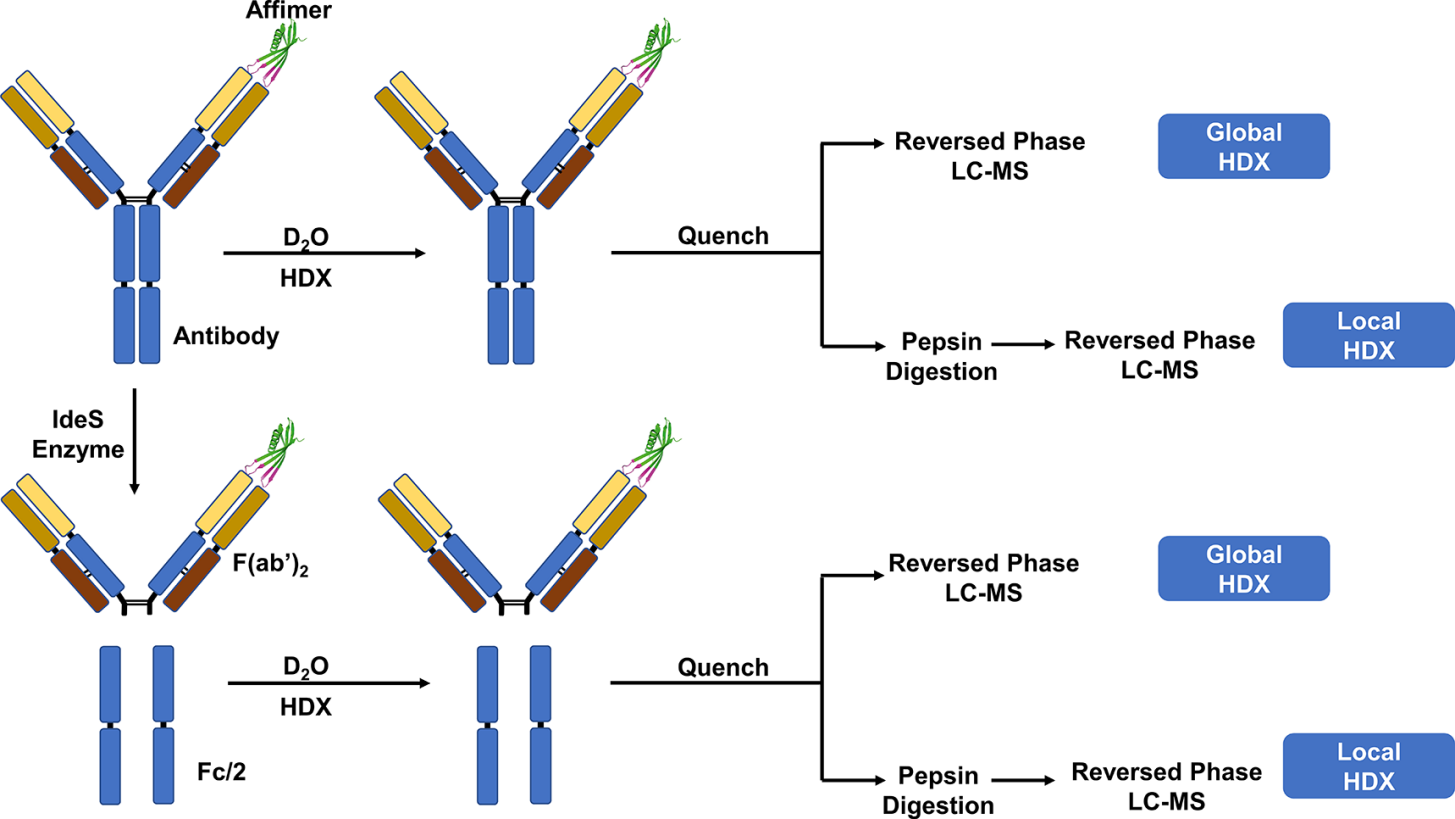
Workflow for the global and local HDX-MS experiments to map affimer–antibody binding sites. Antibody subunits were generated by specific antibody digestion with the IdeS (Fabricator) enzyme before deuteration. All experiments were performed using a liquid handling robot and a cooled LC system (0 °C) after quenching the samples.

In order to reveal the affimer binding sites to trastuzumab and pertuzumab by HDX-MS, global and local HDX analysis strategies were followed as described in Figure 1. Newly developed global HDX-MS workflows involving intact and subunit HDX experiments were initially performed as a screening method to obtain a fast higher-order structure overview and to confirm the formation of a complex between the antibodies and their affimers. Differential deuterium uptake analysis of free and complexed antibodies or their F(ab′)_2_ subunits allowed quick detection of domains with changed HDX behavior. In the next step, the affimer–antibody complexes were investigated by HDX-MS on a local level with the classical bottom-up approach utilizing online pepsin digestion and LC-MS/MS-based peptide analysis.

### HDX-MS of Complexes at the Intact Antibody and Subunit Level

**Figure 2:**
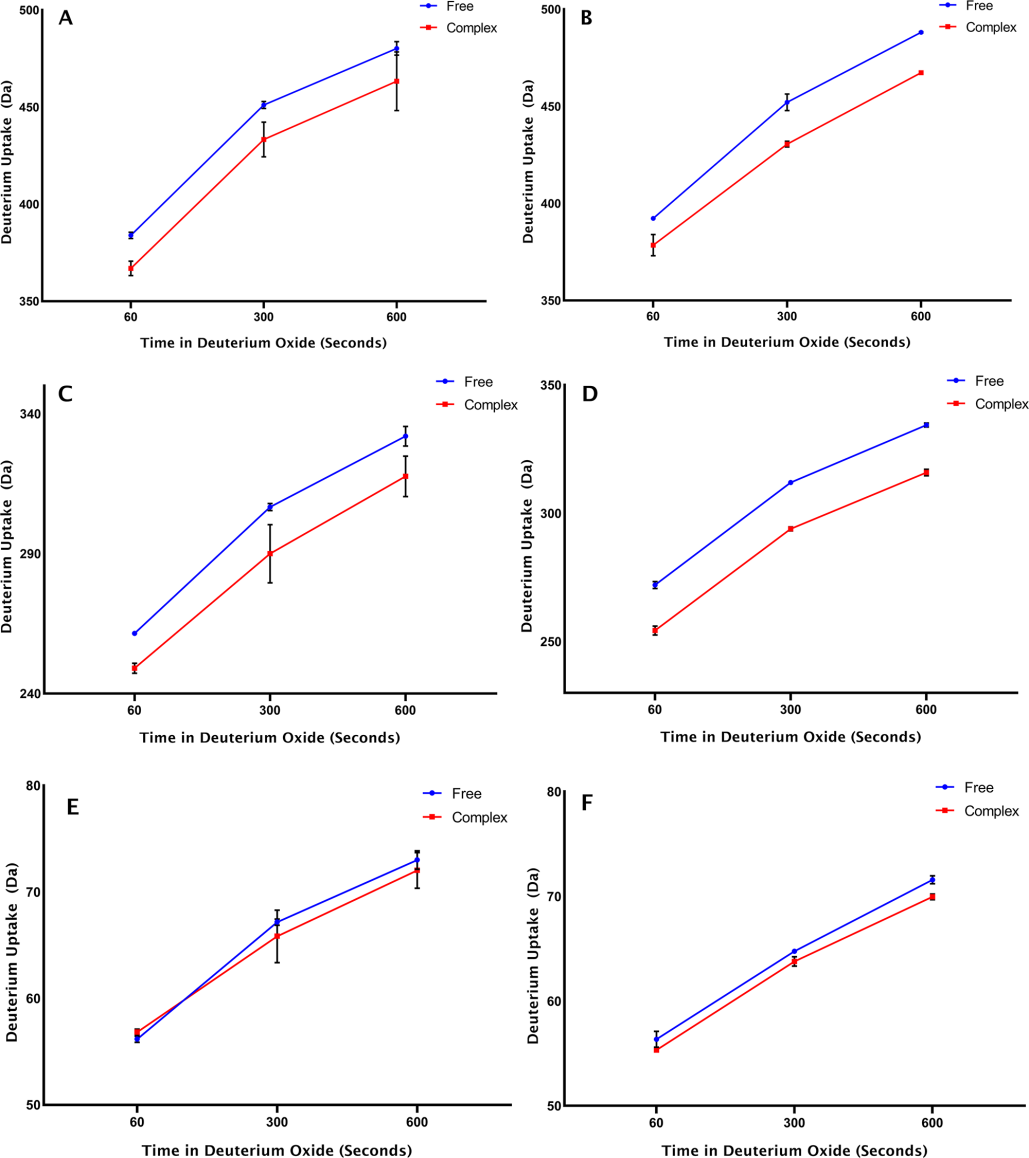
Deuterium uptake over time after comparing free and complexed states of the intact antibody, F(ab′)_2_, and Fc/2 subunit levels of trastuzumab (panels A, C, and E) and pertuzumab (panels B, D, and F), respectively (*n* = 3).

We first tested the applicability of intact antibody HDX-MS workflows to monitor the effects of affimer binding on trastuzumab and pertuzumab deuteration. Intact mAb mass measurement of free antibody and affimer-bound antibody after three deuteration time points revealed a significant reduction in deuterium uptake of 16–17 Da for trastuzumab and 13-21 Da in pertuzumab upon affimer complex formation (Figure 2, Table 1). This protection of antibody structure from HDX correlates with the binding of the affimer protein, which reduces the accessibility of amide nitrogen atoms to deuterium oxide in the protein binding region.

**Table 1:** Average Deuterium Incorporation Difference between Free and Complex States of Trastuzumab and Pertuzumab Measured at the Intact Antibody and Subunit Level^a^

	**Average HDX protection in affimer complex (ΔDa)**		
**Protein form (+glycan)**	**60 s HDX**	**300 s HDX**	**600 s HDX**
Trastuzumab Intact (G0F/G0F)	17.00 (±3.06)	17.77 (±6.16)	16.90 (±8.91)
Trastuzumab F(ab′)_2_	12.47 (±1.32)	16.67 (±6.60)	14.37 (±5.70)
Trastuzumab Fc/2 + G0F	–0.67 (±0.33)	1.33 (±1.36)	0.97 (±1.29)
Pertuzumab Intact (G0F/G0F)	13.77 (±2.99)	21.53 (±3.23)	20.83 (±0.44)
Pertuzumab F(ab′)_2_	17.73 (±1.53)	18.10 (±0.44)	18.50 (±0.50)
Pertuzumab Fc/2 + G0F	1.03 (±0.43)	0.97 (±0.26)	1.63 (±0.37)

a Standard error of mean in brackets.

In order to localize the antibody domain which is binding to the affimer, we monitored HDX in antibody subunits F(ab)2 (100 kDa) and Fc/2 (25 kDa) in the absence or presence of bound affimer. The subunits were generated prior to HDX using the IdeS enzyme, which cuts specifically in the hinge region of IgG1 antibodies.

Figure 2 shows the deuterium uptake over time for free and complexed forms at the intact F(ab′)_2_ and Fc/2 levels of trastuzumab (panels A, C, and E) and pertuzumab (panels B, D, and F), respectively. Table 1 provides the average deuterium uptake differences obtained when the free and complexed states are compared. There is a significant difference in mass between the free and complexed states of intact and F(ab′)_2_ forms of trastuzumab and pertuzumab, which indicates protection of HDX in the antibodies upon Affimer binding. The fact that little or no difference was observed in the Fc/2 regions when comparing the free and complexed states at the subunit level indicates that the Fc/2 regions do not participate in Affimer reagent binding.

### HDX-MS at the Peptide Level

**Figure 3:**
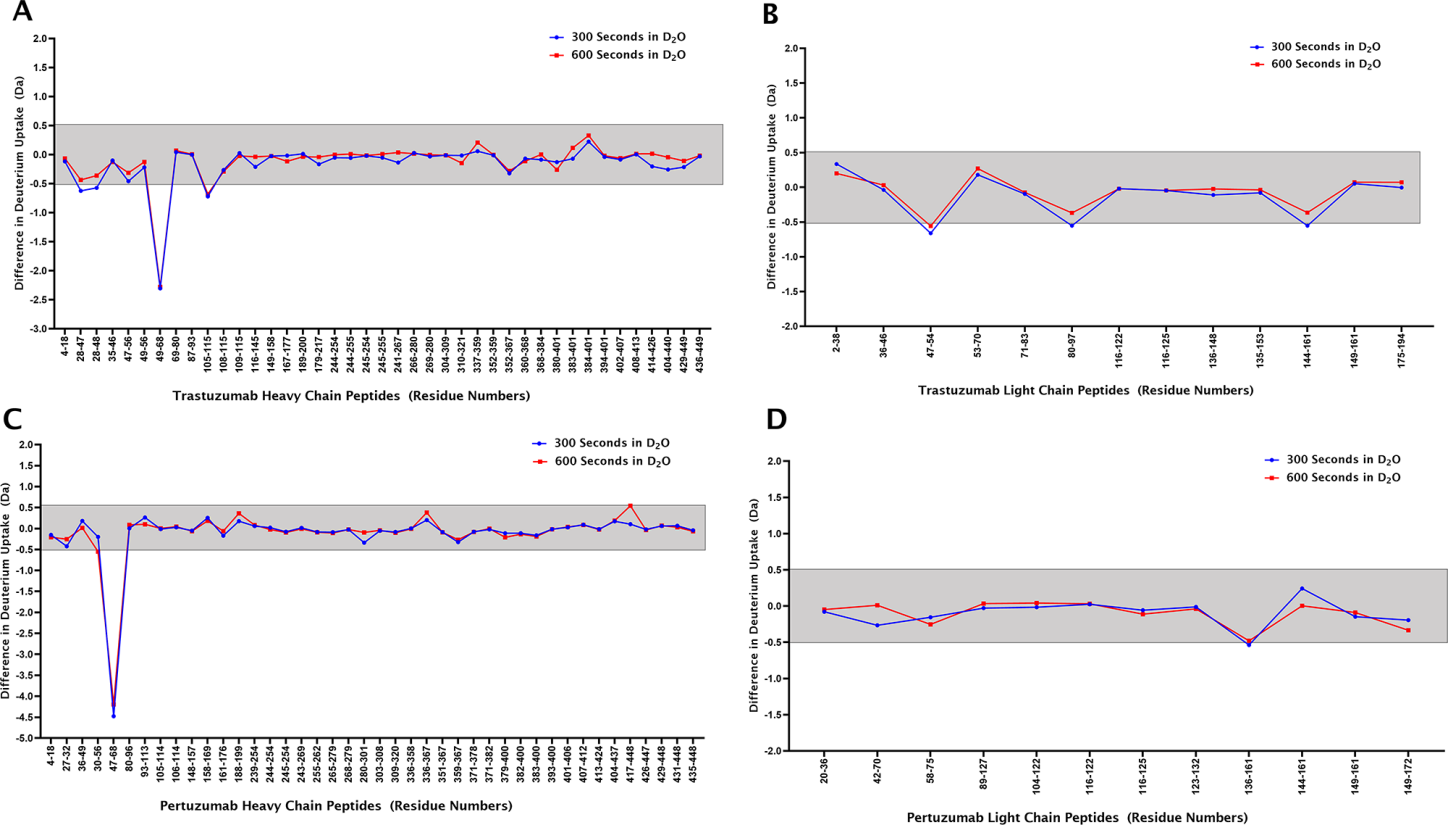
Deuterium difference plots showing the relative deuterium incorporation of the free antibodies vs antibodies in complex with the respective affimers after HDX for 300 and 600 s (5 replicates). Trastuzumab heavy and light chain peptic peptides are shown in panels A and B, and pertuzumab heavy and light chains are shown in panels C and D, respectively. The gray area marks the 0.5 Da confidence interval for calling a significant HDX difference; negative values refer to protection from deuterium incorporation.

For local HDX at the peptide level, similar experiments as at the global level were performed for both antibodies with the inclusion of an online pepsin digestion step before LC-MS analysis, as shown in Figure 1. To determine the deuterium uptake of the different peptides, deuterium difference plots showing the relative deuterium incorporation of the free vs the complexed states of the antibodies after incubation in D_2_O were created. Figure 3 shows the deuterium difference plots for trastuzumab (panels A and B) and pertuzumab (panels C and D). In addition, amino acid sequence heat maps representing the mass differences of the free vs the complexed states of the antibodies after a 600 s incubation in D_2_O were created (Figure S1, Supporting Information). For a peptide to be considered as a site where HDX is significantly changed by complex formation, a threshold value of −0.5 Da mass difference should be observed.^40^ ARIY···GRF (amino acids 49–68 with mass 2335.19 Da) in the complementarity-determining region (CDR) 2 of the trastuzumab heavy chain shows the largest difference indicating that this is part of the binding site for the Affimer reagent. In addition, YAMD···GTL (amino acids 105–115 with mass 1304.56 Da), which has residues that are part of CDR3 of the trastuzumab heavy chain, and LIYSASFL (amino acids 47–54 with mass 913.50 Da), which has residues that are part of CDR2 of the trastuzumab light chain, show mass differences that meet the threshold value (see Figure S1, Supporting Information). This could be due to a conformational change or slight protection provided due to the binding position of the Affimer reagent. Peptides NIKD···LEW (amino acids 28–47 with mass 2411.727 Da) and NIKD···EWV (amino acids 28–48 with mass 2510.859 Da) on the trastuzumab heavy chain and PEDF···PPT (amino acids 80–97 with mass 2162.297 Da) and AKVQ···SQE (amino acids 144–161 with mass 2002.151 Da) on the trastuzumab light chain also achieve the threshold value of −0.5 Da after 300 s of HDX. For pertuzumab, only WVAD···GRF (amino acids 47–68 with mass 2512.25 Da), covering CDR2 of the heavy chain, showed a significant difference indicating that it is part of the binding site for the Affimer reagent (see Figure S1, Supporting Information). Peptide LNNF···SQE (amino acids 136–161 with mass 3036.287 Da) met the threshold value of −0.5 Da after 300 s of HDX. The stacked spectral plots for ARIY···GRF (Figure S2, Supporting Information) and for WVAD···GRF (Figure S3, Supporting Information) show the mass shifts for both peptides for the free (panel A) and complexed (panel B) states after 300 and 600 s of incubation in D_2_O indicating that HDX is almost complete after 300 s. The deuterium uptake plots for the peptides ARIY···GRF (panel A) and WVAD···GRF (panel B) also show the mass difference when the free and complexed states are compared at 300 and 600 s incubation in D_2_O (Figure S4, Supporting Information).

### Modeling of Binding Interaction

**Figure 4:**
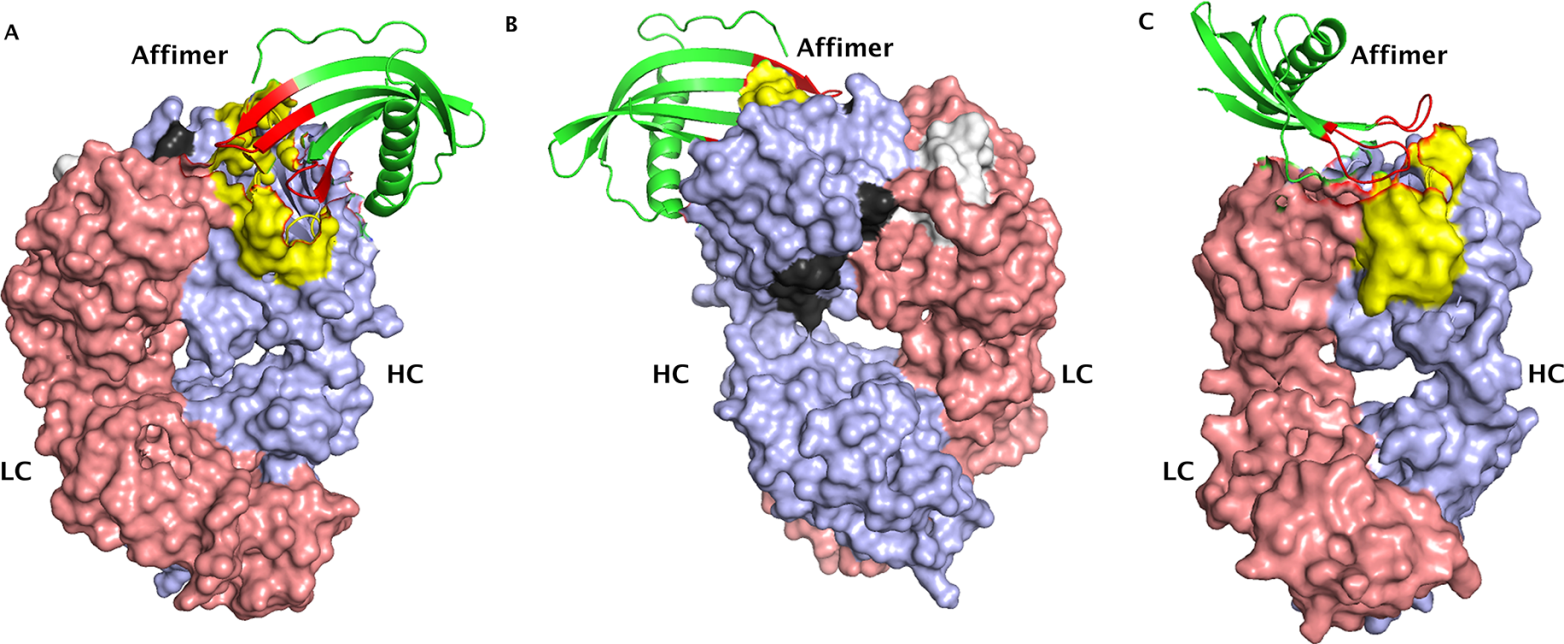
Binding interaction models for trastuzumab (panel A – front view and panel B – back view) and pertuzumab Fab (panel C) with their respective Affimer reagents. The heavy chains (HC) for both antibodies are colored in lilac, the light chains (LC) are colored in pink, and the interacting peptides are colored in yellow. The two trastuzumab peptides that met the threshold value at 300 and 600 s of HDX are depicted in black (YAMD···GTL) and white (LIYSASFL). The binding loops of the Affimer reagents are colored in red, and the backbone is colored in green (ribbon structure). Trastuzumab and pertuzumab F(ab′)_2_ structures were obtained from PDB, accession number 6OGE. The affimer structure was also obtained from PDB, accession number 5ML9.

Binding interactions between the antibodies and Affimer reagents were created using computer-based models based on the encoding of information obtained from known or predicted protein interfaces. The heavy chain peptides ARIY···KGRF (trastuzumab) and WVAD···GRF (pertuzumab) along with the residues in the binding loops of their respective Affimer reagents were listed as active residues that are directly involved in the interaction. Figure 4 shows the models obtained for trastuzumab with its Affimer reagent (panels A and B) and pertuzumab with its Affimer reagent (panel C). The models shown here were selected based on the best scores for the van der Waals intermolecular energy, electrostatic intermolecular energy, desolvation energy, and binding energy. The overall Z-score describes how much the standard deviation of one particular model deviates from the average of all the models.

## Discussion and Conclusion

HDX-MS has been used to characterize the binding sites of specific Affimer reagents to the therapeutic, monoclonal antibodies trastuzumab and pertuzumab. Significant mass differences between complexed and free forms indicated that certain regions of the antibodies are protected from HDX due to the binding of the Affimer reagents. Both Affimer reagents bind to the CDR2 regions of the heavy chains of trastuzumab and pertuzumab, respectively. This likely forms the basis for the specificity of the Affimer reagents. CDR2 on the heavy chain is longer than CDRs 1 and 3, which may have facilitated selecting specific Affimer reagents by phage display. Interestingly, CDRs on the light chain do not seem to contribute to binding. The Fc/2 regions of the antibodies did not show significant mass differences upon HDX, indicating that they are not involved in the binding process. This was expected, since these regions are identical between trastuzumab and pertuzumab, and affimer selection was based on phage display using the Fab part of the antibodies only.

In light of using these Affimer reagents for *in vivo* biotransformation studies in breast cancer patients,^41^ it is important to know whether the binding sites contain residues that may be prone to modifications.^42^ As mentioned earlier, modifications in a binding site could cause a binder to not recognize its target. This could result in the Affimer reagents not capturing certain variants of trastuzumab or pertuzumab and thereby providing an incomplete picture. However, the Affimer reagents were able to bind all the trastuzumab and pertuzumab variants observed in the *in vivo* biotransformation study suggesting that none of the modifications occurring in the variants affected the binding sites. Although the deamidation of an asparagine residue (N55) in the binding site of a trastuzumab variant was described, it did not prevent recognition by the Affimer reagents. This is interesting because it was the same deamidation that resulted in the inability of an anti-idiotypic antibody to bind to trastuzumab as described by Bults et al.^36^ In fact, our HDX-MS data from overlapping trastuzumab H-CDR2 peptides clearly indicate that Affimer binding occurs within the peptide stretch 57–68, because peptide 49–56 containing N55 displays no signifcant change in HDX upon complex formation.

The observation that certain other groups of residues in trastuzumab show notable mass differences upon HDX when in the complexed or free states indicates that binding the affinity reagent induces conformational changes. This may make defining the actual binding site by HDX-MS somewhat ambiguous, since it is not possible to discriminate between residues that are involved in binding and those that change their HDX characteristics due to a conformational change.

The HDX data were used to build structural models of the Affimer reagent–antibody interactions. While these models show the interaction between the variable loops on the Affimer reagents and the CDR2 regions of the heavy chains of trastuzumab and pertuzumab, respectively, they must be taken with caution, since alternative models are possible albeit with less favorable parameter scores. More detailed models of the complexes require data at atomic resolution as provided by X-ray crystallography.

## Supporting Information Available

**Figure S1**: Amino acid sequence heat maps showing peptide mass differences of the free vs the complexed states after incubation in D_2_O for 600 s for trastuzumab (panel A) and pertuzumab (panel B), respectively (lines below the numbers represent the identified peptides). **Figure S2**: Stacked spectral plot for ARIYPTNGYTRYADSVKGRF (trastuzumab) showing the corresponding mass shift. Free state (panel A) and complex state (panel B). In both panels, the lowest three spectra – controls, the middle five spectra – 300 s in D_2_O, and the topmost five spectra – 600 s in D_2_O (asterisk represents the monoisotopic peak). **Figure S3**: Stacked spectral plot for WVADVNPNSGGSIYNQRFKGRF (pertuzumab) showing the corresponding mass shift. Free state (panel A) and complex state (panel B). In both panels, the lowest three spectra – controls, the middle five spectra – 300 s in D_2_O, and the topmost five spectra – 600 s in D_2_O (asterisk represents the monoisotopic peak). **Figure S4**: Deuterium uptake plots for the peptides ARIYPTNGYTRYADSVKGRF from trastuzumab (panel A) and WVADVNPNSGGSIYNQRFKGRF from pertuzumab (panel B) showing the mass differences when the free and complexed states are compared at 300 and 600 s incubation in D_2_O (*n* = 5) (PDF)
Trastuzumab and pertuzumab peptide lists (XLSX)
