# Supplementary material for: Determination of Binding Sites on Trastuzumab and Pertuzumab to Selective Affimers Using Hydrogen–Deuterium Exchange Mass Spectrometry
Source: J Am Soc Mass Spectrom. 2023 Mar 24;34(4):775–83. doi: 10.1021/jasms.3c00069 (PMC10080681; doi:10.1021/jasms.3c00069)
Supplement: Supplementary file 1 — js3c00069_si_001.pdf [file js3c00069_si_001.pdf]

Supporting Information

Determination of Binding Sites on Trastuzumab and Pertuzumab to Selective Affimers Using Hydrogen–Deuterium Exchange Mass Spectrometry

Oladapo Olaleye[1], Christian Graf[2], Baubek Spanov[1], Natalia Govorukhina[1], Matthew R. Groves[3], Nico C. van de Merbel[1,4], Rainer Bischoff[1]*

[1]Analytical Biochemistry, Department of Pharmacy, University of Groningen, A. Deusinglaan 1, 9713 AV Groningen, The Netherlands

[2]Novartis Technical Research & Development Biologics, Hexal AG, Keltenring 1+3, 82041 Oberhaching, Germany

[3]Drug Design, Department of Pharmacy, University of Groningen, A. Deusinglaan 1, 9713 AV, Groningen, The Netherlands

[4]ICON Bioanalytical Laboratories, Amerikaweg 18, 9407, TK Assen, The Netherlands

*Corresponding author: r.p.h.bischoff@rug.nl

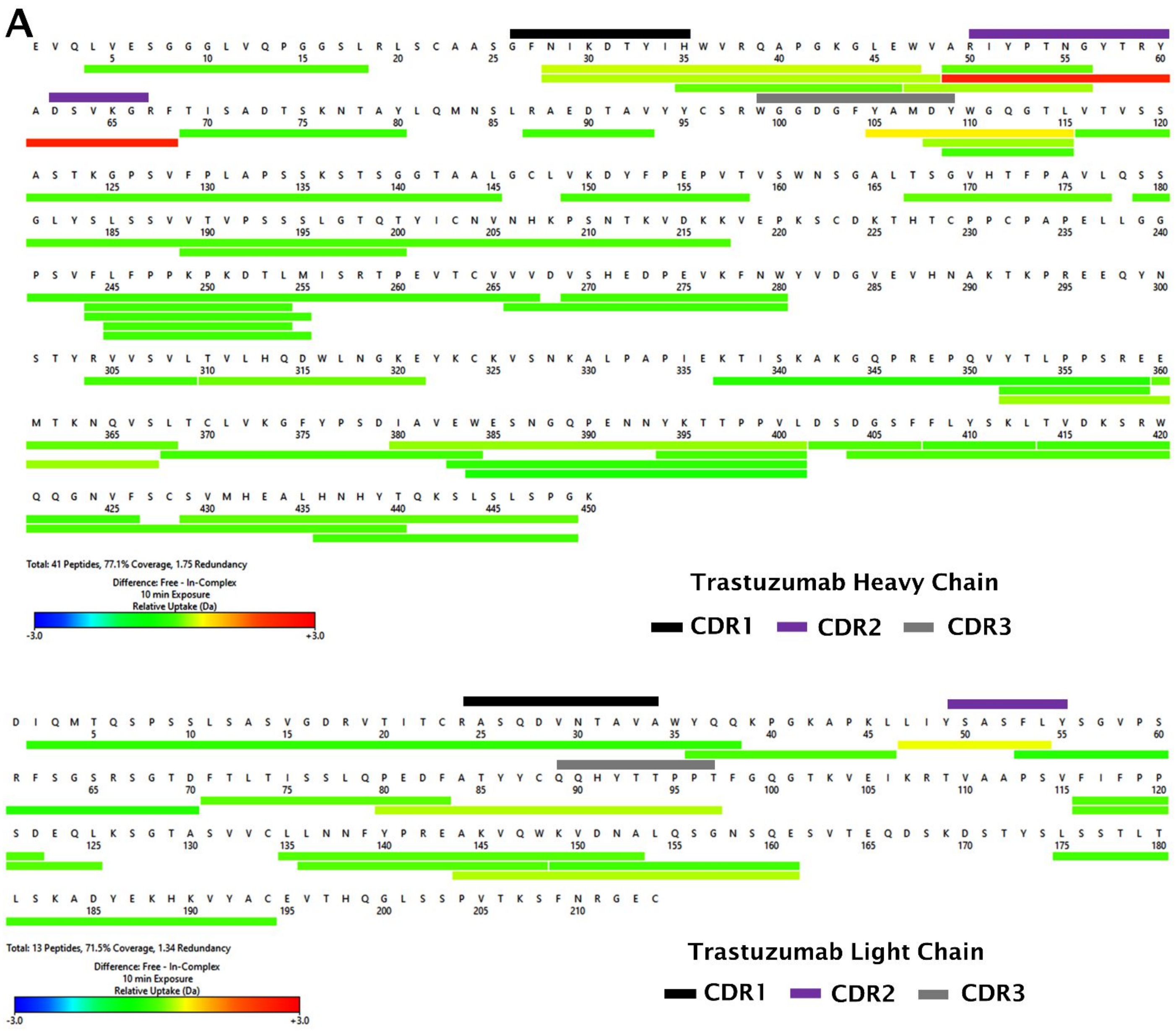
A
EVQLVESGGGLVQPGGSLRLSCAASGFNIKDTYIHWVRQAPGKGLEWVARIYPTNGYTRY
ADSVKGRFTISADTSKNTAYLQMNSLRAEDTAVYYCSRWGGDGFYAMDYWGQGTLVTVSS
ASTKGPSVFPLAPSSKSTSGGTAALGCLVKDYFPEPVTVSWNSGALTSGVHTFPAVLQSS
GLYSLSSVVTVPSSSLGTQTYICNVNHKPSNTKVDKKVEPKSCDKTHTCPPCPAPELLGG
PSVFLFPPKPKDTLMISRTPEVTCVVVDVSHEDPEVKFNWYVDGVEVHNAKTKPREEQYN
STYRVVSVLTVLHQDWLNGKEYKCKVSNKALPAPIEKTISKAKGQPREPQVYTLPPSREE
MTKNQVSLTCLVKGFYPSDIAVEWESNGQPENNYKTTPPVLDSDGSFFLYSKLTVDKSRW
QQGNVFSCSVMHEALHNHYTQKSLSLSPGK
Total: 41 Peptides, 77.1% Coverage, 1.75 Redundancy
Difference: Free - In-Complex
10 min Exposure
Relative Uptake (Da)
-3.0
+3.0
Trastuzumab Heavy Chain
CDR1
CDR2
CDR3
DIQMTQSPSSLSASVGDRVTITCRASQDVNTAVAWYQQKPGKAPKLLIYSASFLYSGVPS
RFSGSRSGTDFTLTISSLQPEDFATYYCQQHYTTPPTFGQGTKVEIKRTVAAPSVFIFPP
SDEQLKSGTASVVCLLNNFYPREAKVQWKVDNALQSGNSQESVTEQDSKDSTYSLSSTLT
LSKADYEKHKVYACEVTHQGLSSPVTKSFNRGEC
Total: 13 Peptides, 71.5% Coverage, 1.34 Redundancy
Difference: Free - In-Complex
10 min Exposure
Relative Uptake (Da)
-3.0
+3.0
Trastuzumab Light Chain
CDR1
CDR2
CDR3

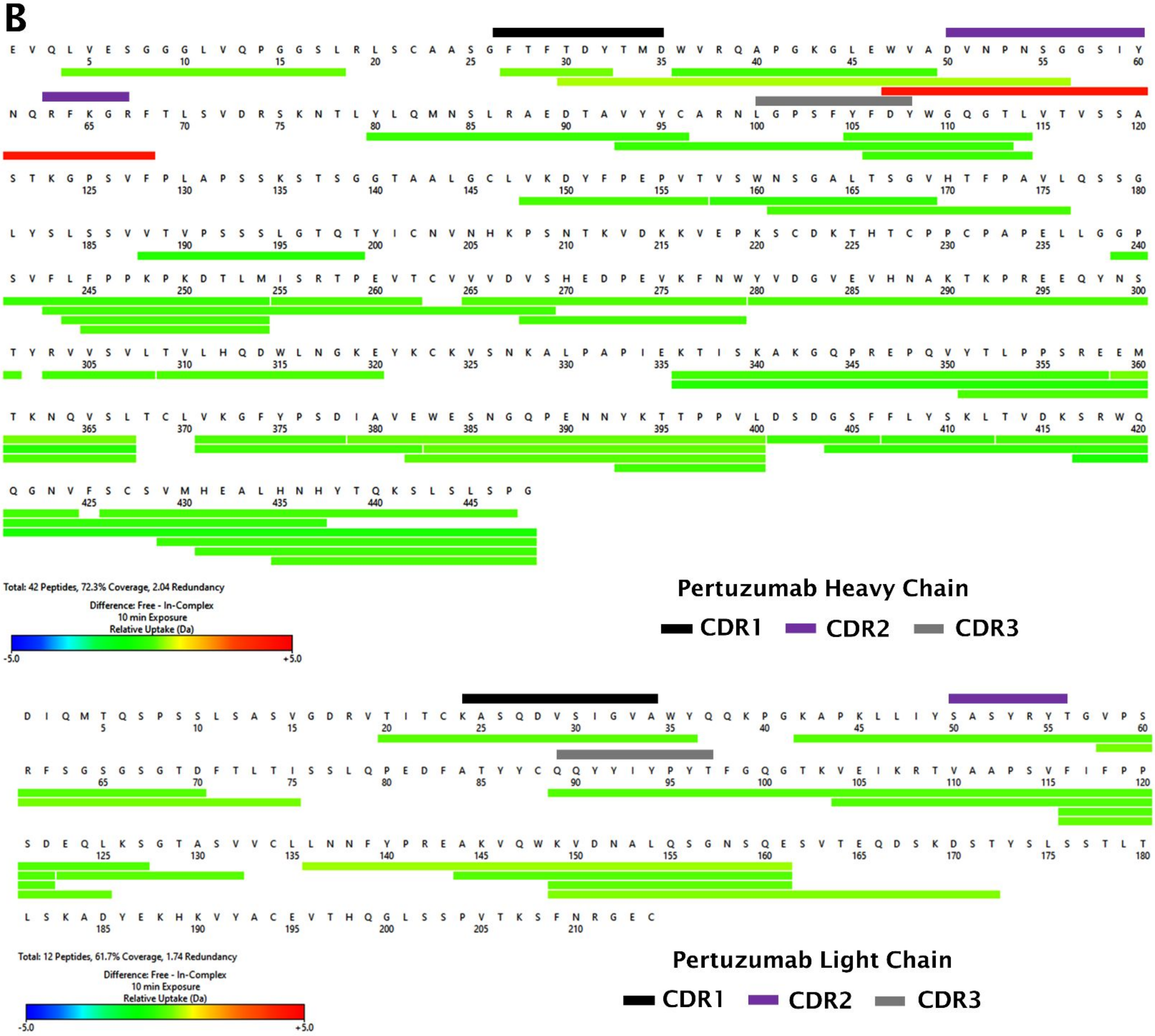

Figure S1: Amino acid sequence heat maps showing peptide mass differences of the free vs the complexed states after incubation in D_2O for 600secs for trastuzumab (panel A) and pertuzumab (panel B), respectively (lines below the numbers represent the identified peptides).

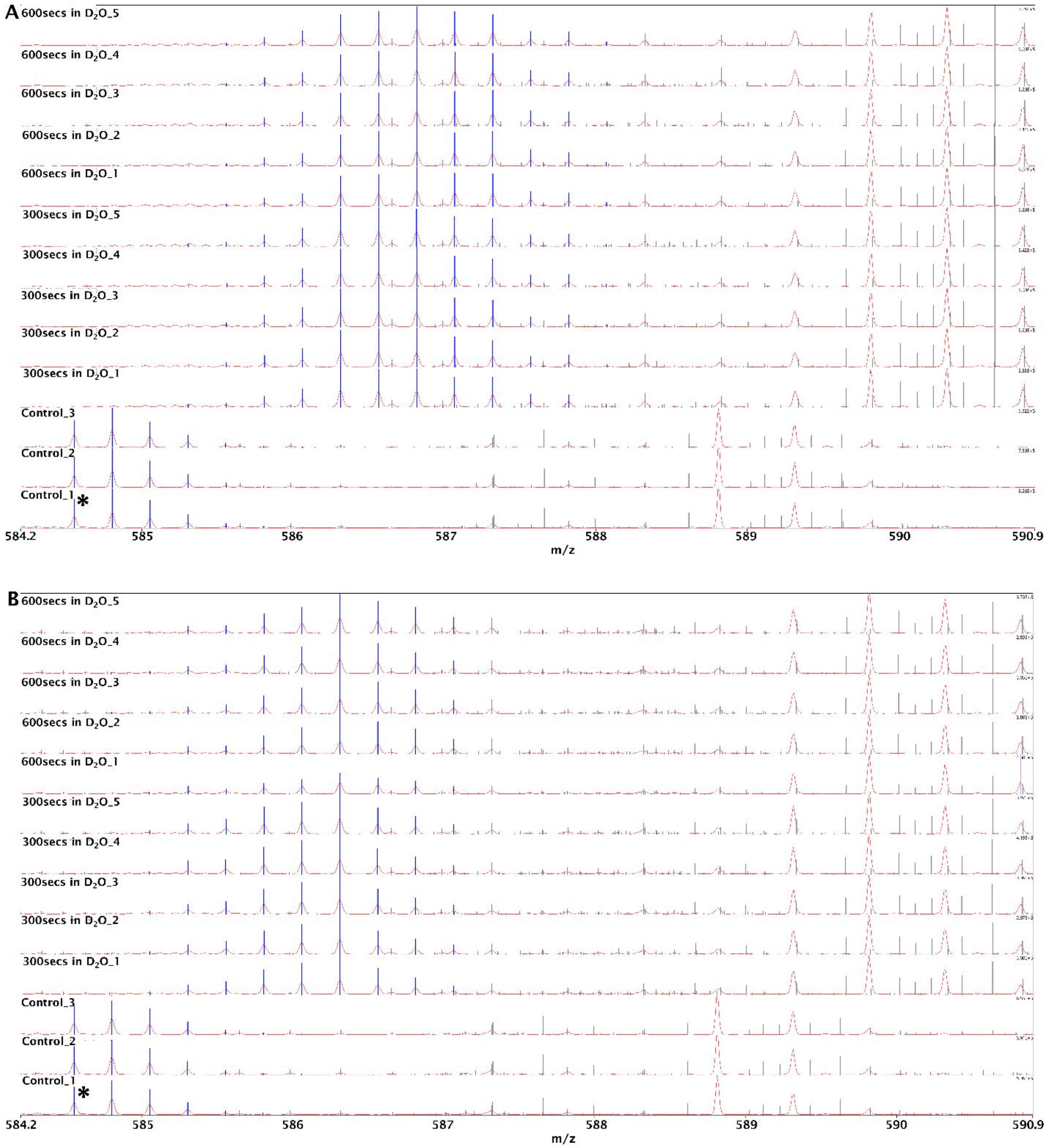

Figure S2: Stacked spectral plot for ARIYPTNGYTRYADSVKGRF (trastuzumab) showing the corresponding mass shift. Free state (panel A) and complex state (panel B). In both panels, the lowest 3 spectra – controls, the middle 5 spectra – 300secs in D_2O and the topmost 5 spectra – 600secs in D_2O (asterisk represents the monoisotopic peak).

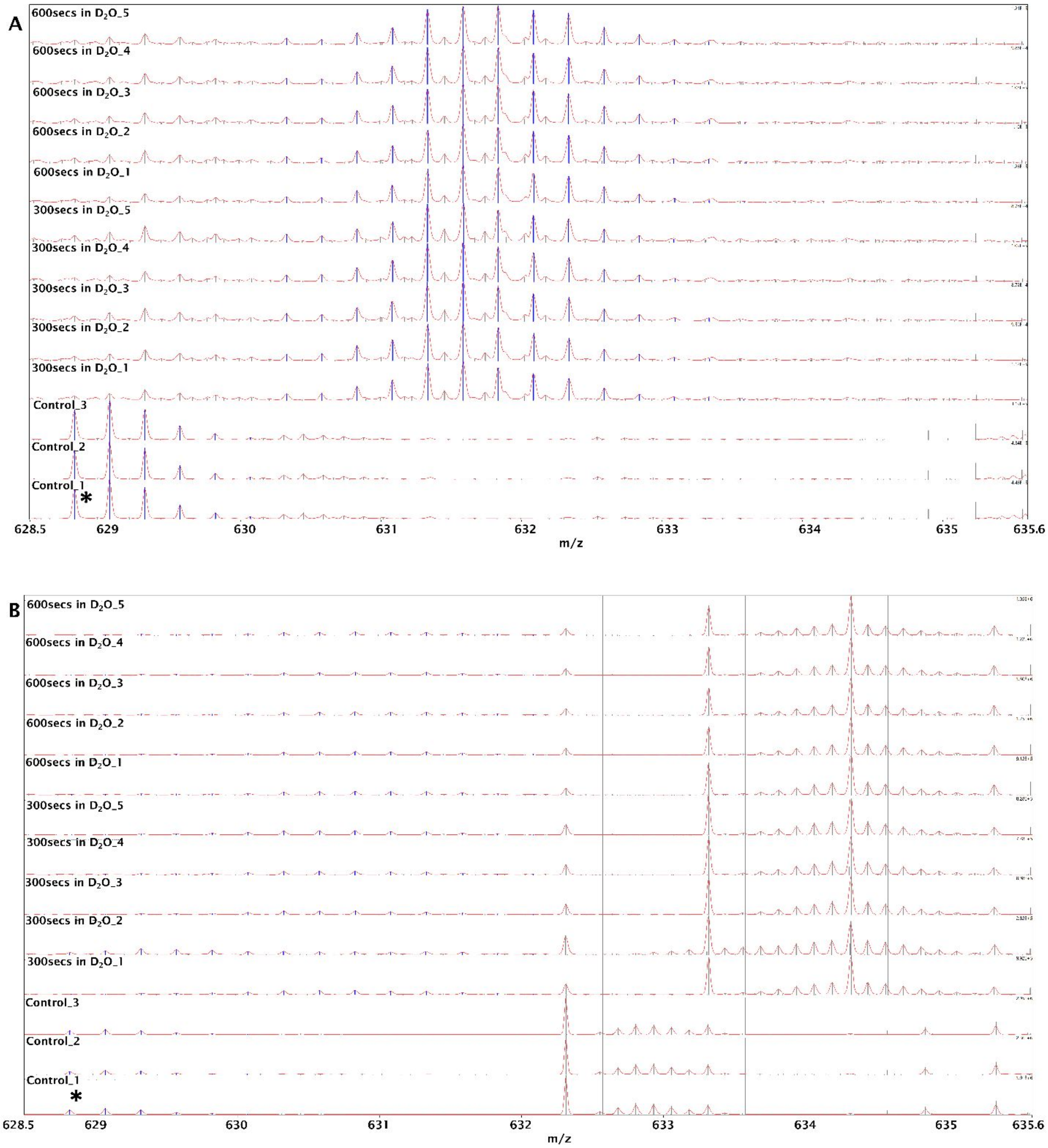

Figure S3: Stacked spectral plot for WVADVNPNSGGSIYNQRFKGRF (pertuzumab) showing the corresponding mass shift. Free state (panel A) and complex state (panel B). In both panels, the lowest 3 spectra – controls, the middle 5 spectra – 300secs in D_2O and the topmost 5 spectra – 600secs in D_2O. (asterisk represents the monoisotopic peak).

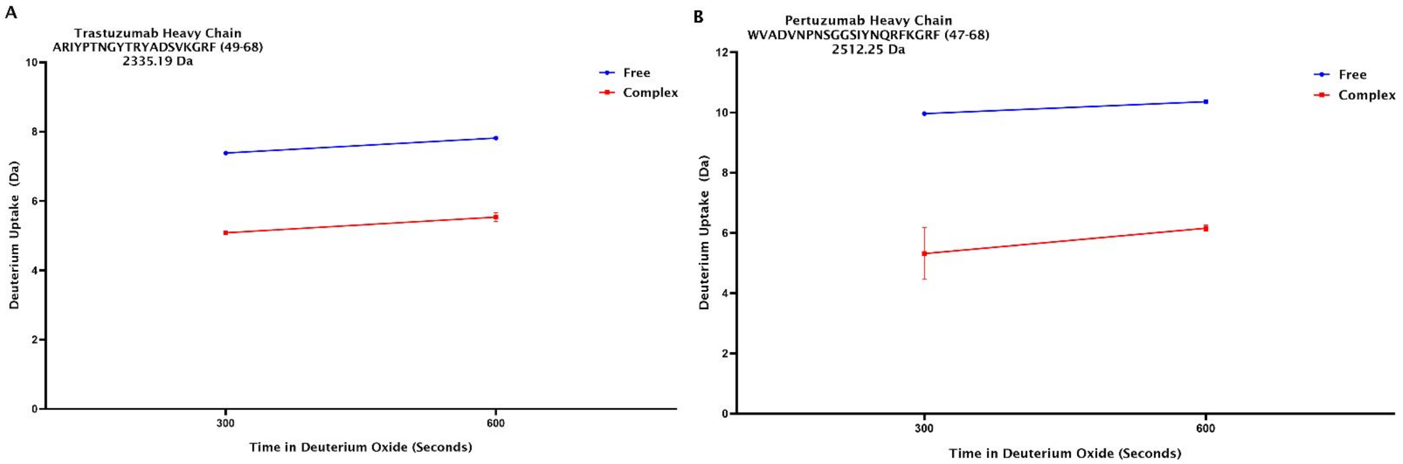

Figure S4: Deuterium uptake plots for the peptides ARIYPTNGYTRYADSVKGRF from trastuzumab (panel A) and WVADVNPNSGGSIYNQRFKGRF from pertuzumab (panel B) showing the mass differences when the free and complexed states are compared at 300 and 600secs incubation in D_2O (n=5).
